# Supplementary material for: A modular neural circuit for computing the motion of objects
Source: bioRxiv. 2026 Jun 8:2026.06.07.730718. Preprint. [Version 1] doi: 10.64898/2026.06.07.730718 (PMC13278120; doi:10.64898/2026.06.07.730718)
Supplement: Supplementary file 1 [file NIHPP2026.06.07.730718v1-supplement-1.pdf]

bioRxiv preprint doi: https://doi.org/10.64898/2026.06.07.730718; this version posted June 8, 2026. The copyright holder for this preprint (which was not certified by peer review) is the author/funder, who has granted bioRxiv a license to display the preprint in perpetuity. It is made available under a CC-BY 4.0 International license.

Supplementary Information

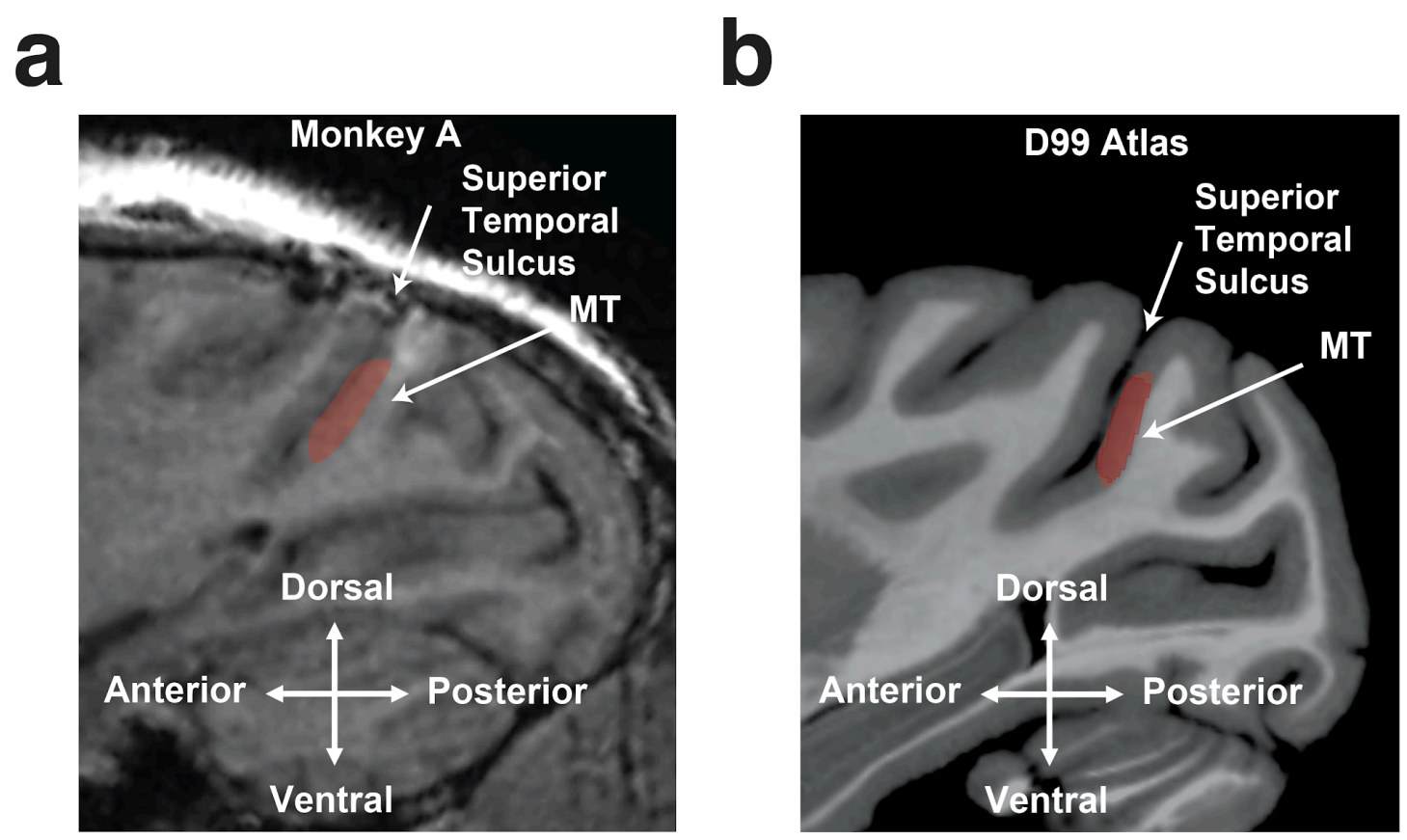

Supplementary Figure 1. MR image of the superior temporal sulcus and area MT in monkey A and the D99 atlas. Sagittal MR image from monkey A (a) and the D99 macaque brain atlas (b). The location of the superior temporal sulcus and area MT (red shaded) are illustrated.

bioRxiv preprint doi: https://doi.org/10.64898/2026.06.07.730718; this version posted June 8, 2026. The copyright holder for this preprint (which was not certified by peer review) is the author/funder, who has granted bioRxiv a license to display the preprint in perpetuity. It is made available under a CC-BY 4.0 International license.

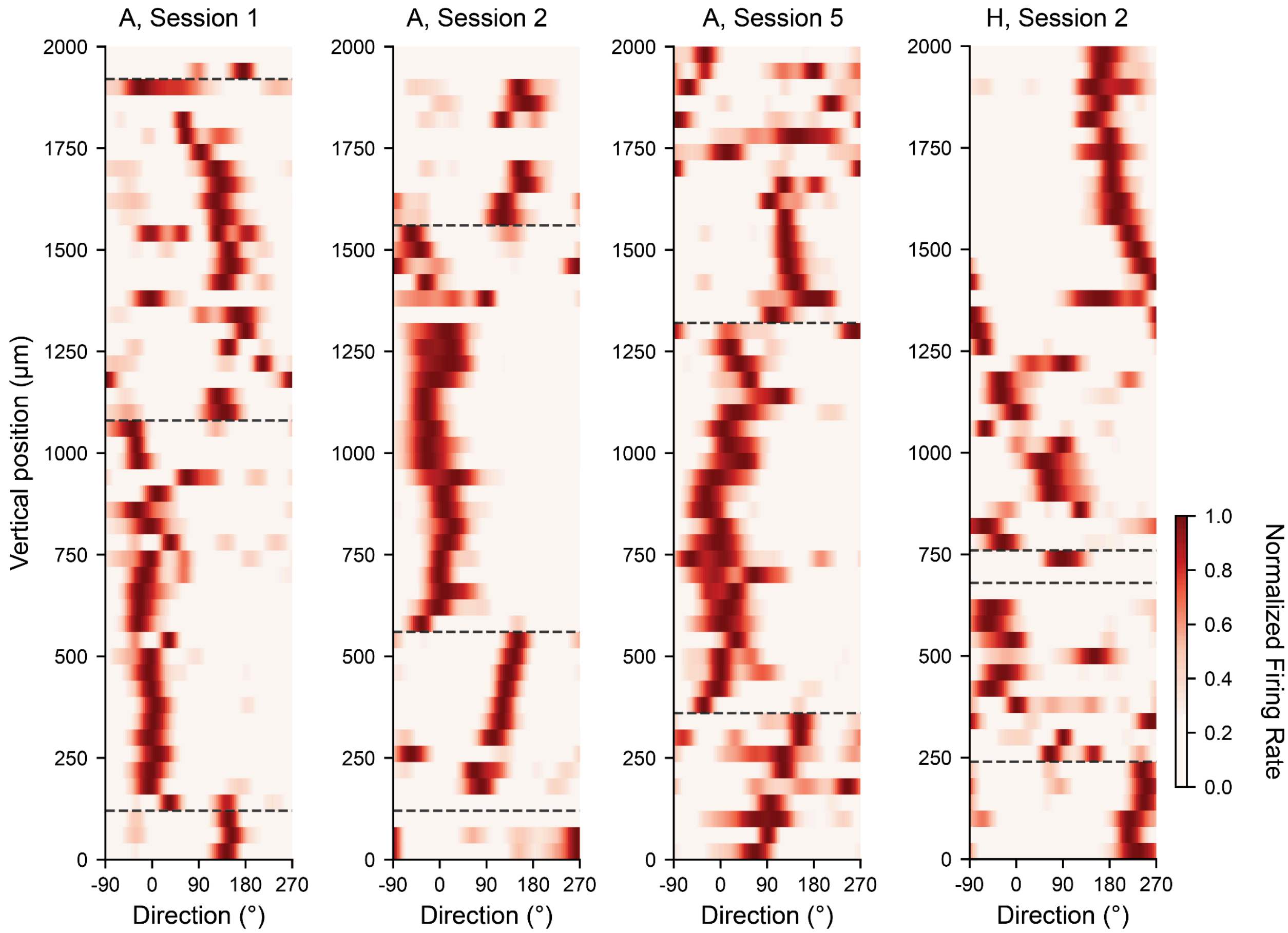

Supplementary Figure 2. Additional examples of modular organization of direction tuning in area MT. Grating direction tuning curves for all neurons at positions relative to the probe tip in 40 μm bins in four additional example sessions. Conventions follow **Fig. 1b**.

bioRxiv preprint doi: https://doi.org/10.64898/2026.06.07.730718; this version posted June 8, 2026. The copyright holder for this preprint (which was not certified by peer review) is the author/funder, who has granted bioRxiv a license to display the preprint in perpetuity. It is made available under a CC-BY 4.0 International license.

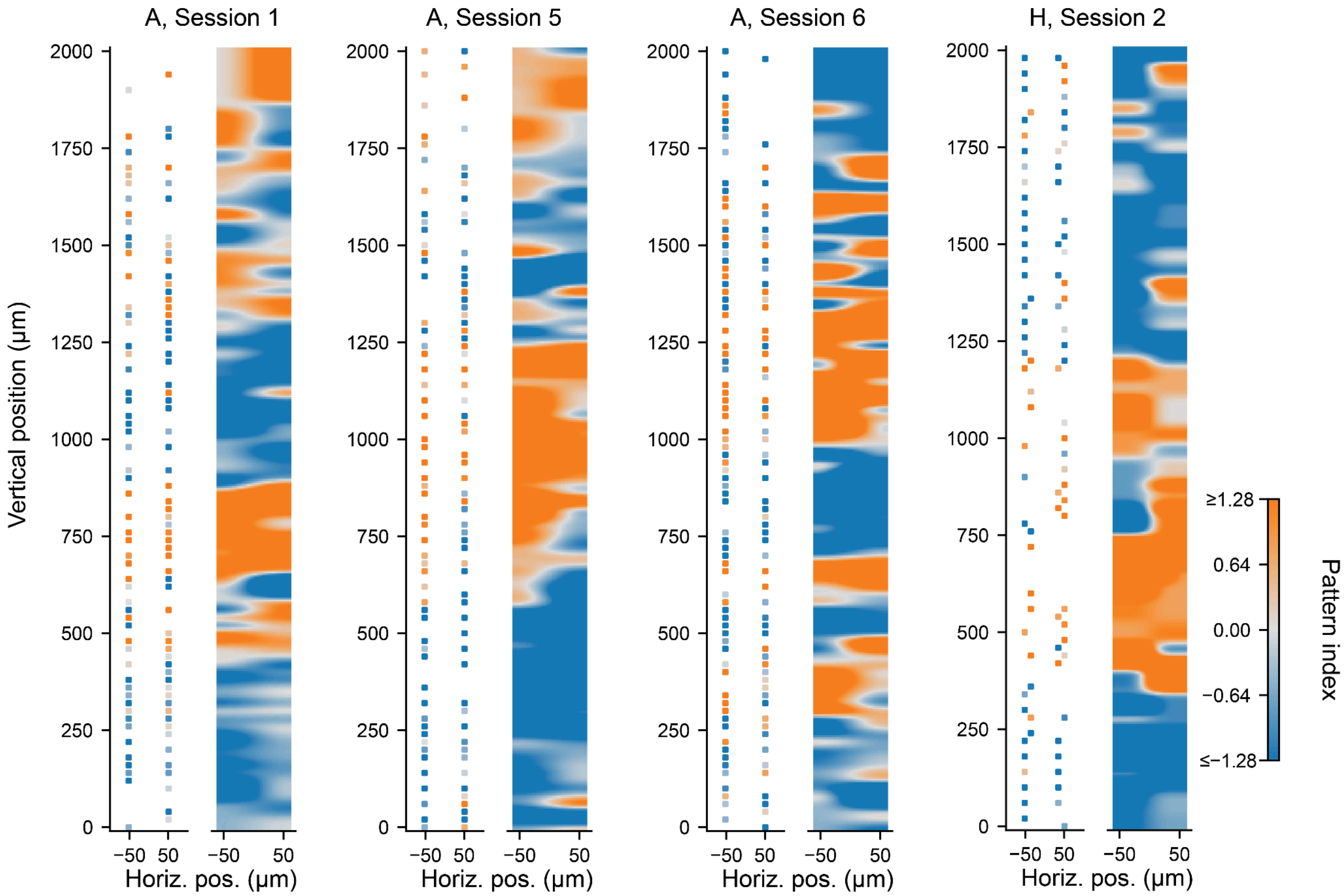

Supplementary Figure 3. Additional examples of modular organization of pattern and component neurons. Pattern index for all neurons at positions relative to the probe tip in four additional example sessions. Conventions follow **Fig. 3b**.

bioRxiv preprint doi: https://doi.org/10.64898/2026.06.07.730718; this version posted June 8, 2026. The copyright holder for this preprint (which was not certified by peer review) is the author/funder, who has granted bioRxiv a license to display the preprint in perpetuity. It is made available under a CC-BY 4.0 International license.

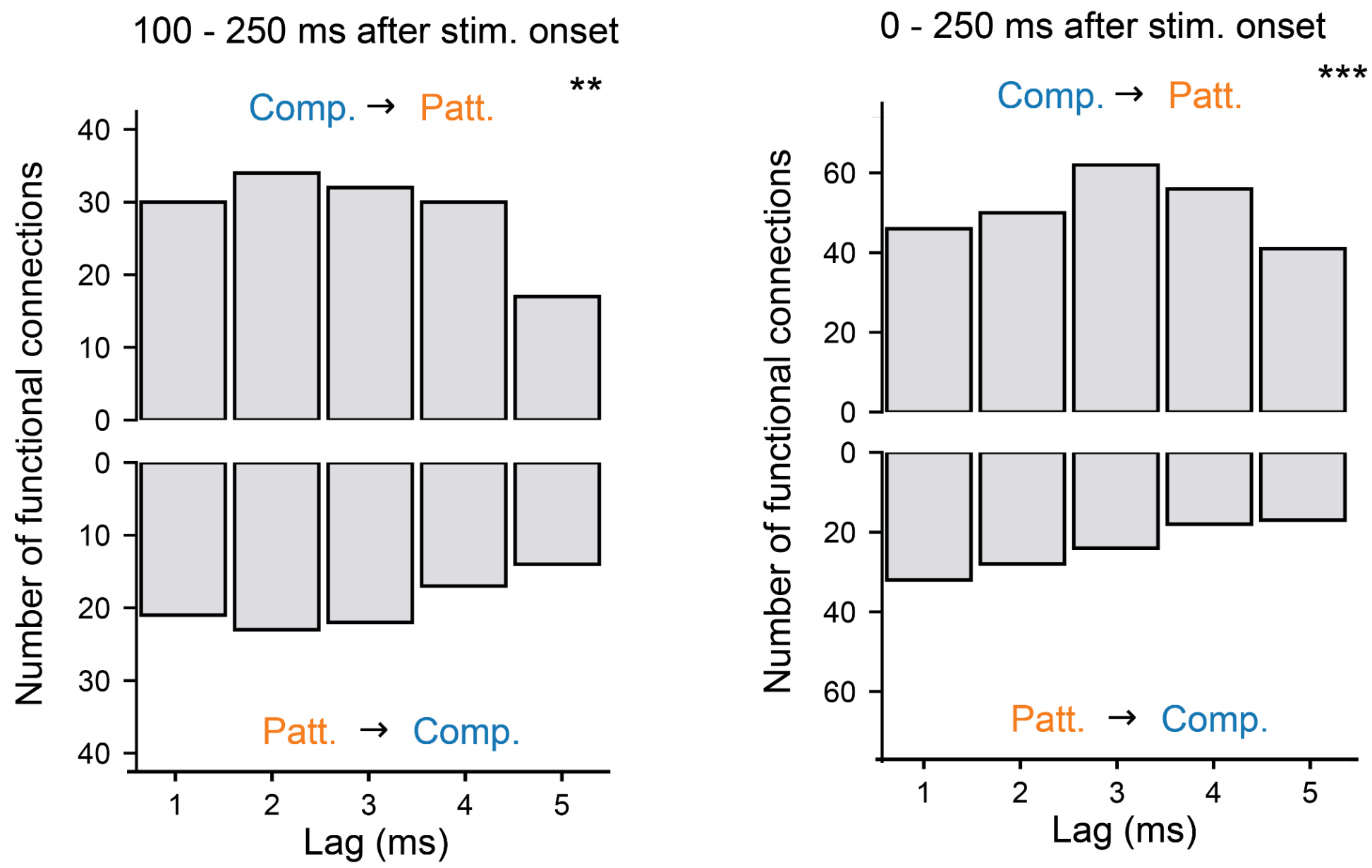

Supplementary Figure 4. Functional connections between component and pattern neurons, computed excluding or including the early visual period. Functional connection asymmetry is robust to the inclusion of the early visual period. The distribution of lags for component-to-pattern and pattern-to-component functional connections is illustrated across all sessions. Functional connectivity is computed using the time interval from 100 ms - 250 ms after stimulus onset (left) and 0 ms - 250 ms after stimulus onset (right). Conventions follow **Fig. 5b**.

bioRxiv preprint doi: https://doi.org/10.64898/2026.06.07.730718; this version posted June 8, 2026. The copyright holder for this preprint (which was not certified by peer review) is the author/funder, who has granted bioRxiv a license to display the preprint in perpetuity. It is made available under a CC-BY 4.0 International license.

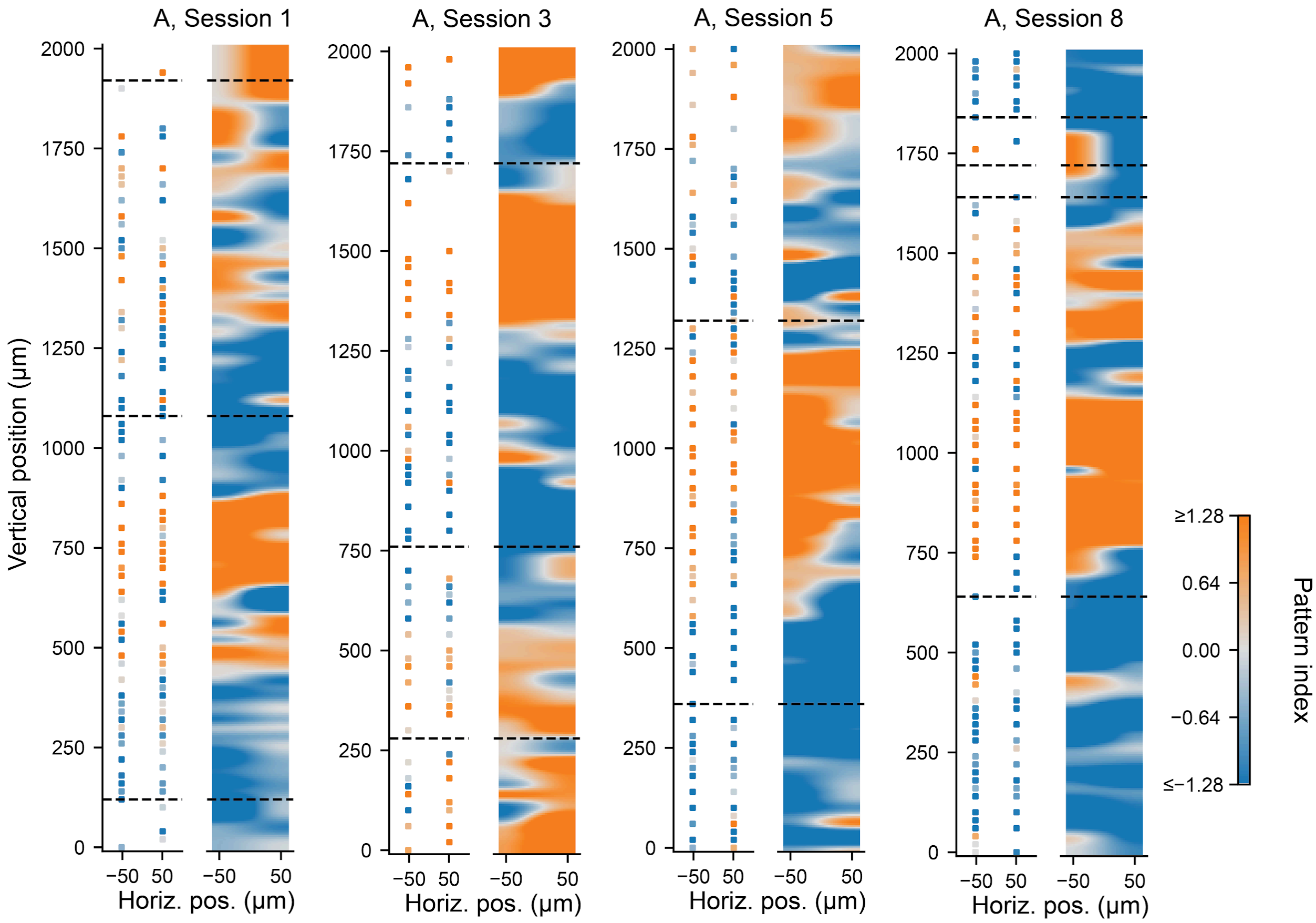

Supplementary Figure 5. Additional examples of organization of component and pattern neurons with respect to the cortical map for motion direction. Reversals (dashed black lines) overlaid on the map of pattern index for all neurons at positions relative to the probe tip in four additional example sessions. Conventions follow **Fig. 6b**.

bioRxiv preprint doi: https://doi.org/10.64898/2026.06.07.730718; this version posted June 8, 2026. The copyright holder for this preprint (which was not certified by peer review) is the author/funder, who has granted bioRxiv a license to display the preprint in perpetuity. It is made available under a CC-BY 4.0 International license.

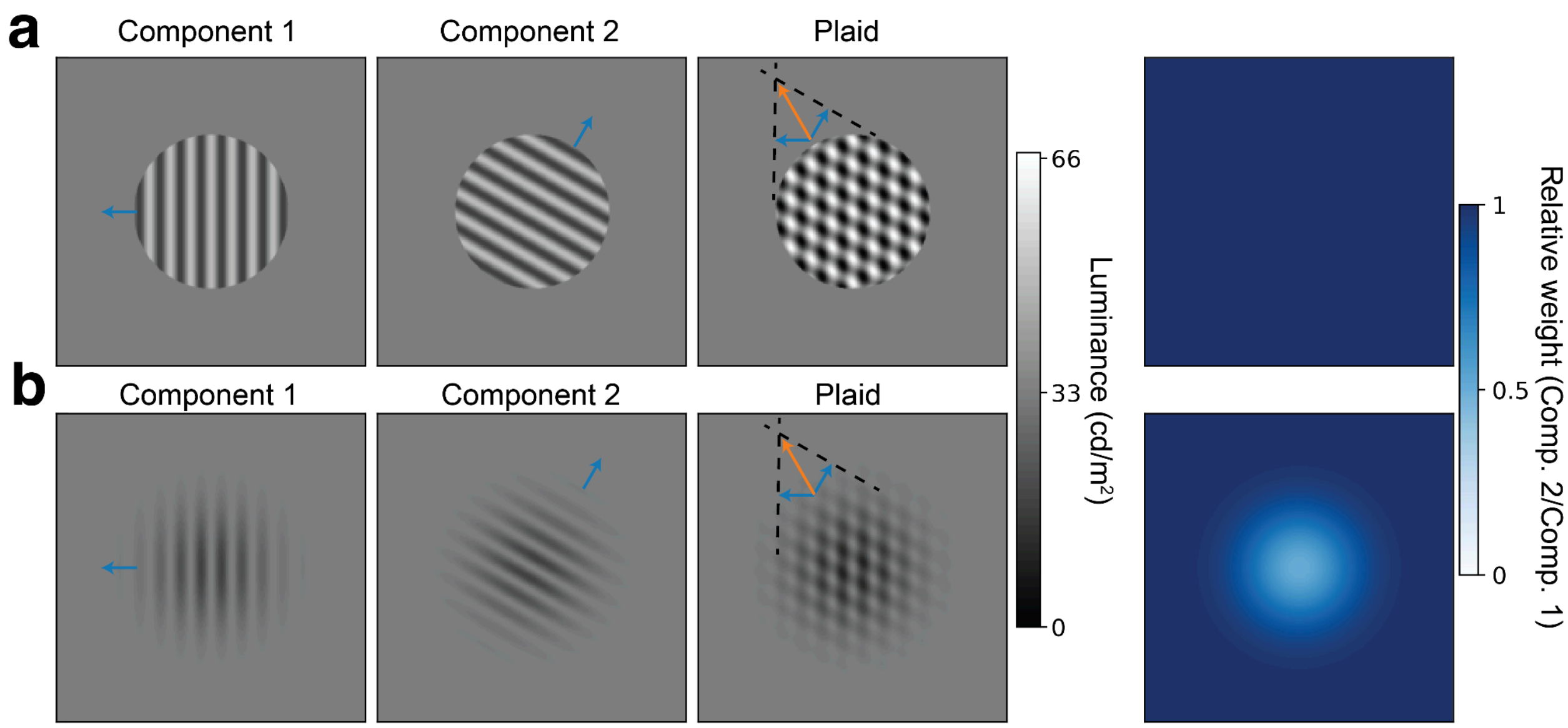

Supplementary Figure 6. Grating and plaid stimuli for measuring pattern and component motion selectivity. (**a**) First plaid stimulus set (A: Sessions 1-5, H: Session 1). Two example component gratings, offset by 120°, and corresponding plaid stimulus constructed by superimposing the two components with equal weights. The blue arrows denote the component motion speed and drift direction. The dashed lines denote the constraints induced by component motion, and the orange arrow denotes the pattern motion vector derived from the intersection of constraints model. (**b**) Second plaid stimulus set (A: Sessions 6-13, H: Session 2-4). Two example component gratings, offset by 120°, and corresponding plaid stimulus constructed by superimposing the two components with the relative weights of the components derived from a Gaussian envelope that varies between 0.5 and 1. Conventions are similar to (a). All plaid stimuli used in this study can be described as a weighted sum of the two components, $P = (C_1 - \beta) + \alpha(C_2 - \beta) + \beta$, where β is the background luminance, C_1 and C_2 are the two components, and α is the relative weight of C_2 to C_1. In the first stimulus set (a), α is set to unity, and in the second stimulus set (b), α is determined by a Gaussian envelope.

bioRxiv preprint doi: https://doi.org/10.64898/2026.06.07.730718; this version posted June 8, 2026. The copyright holder for this preprint (which was not certified by peer review) is the author/funder, who has granted bioRxiv a license to display the preprint in perpetuity. It is made available under a CC-BY 4.0 International license.

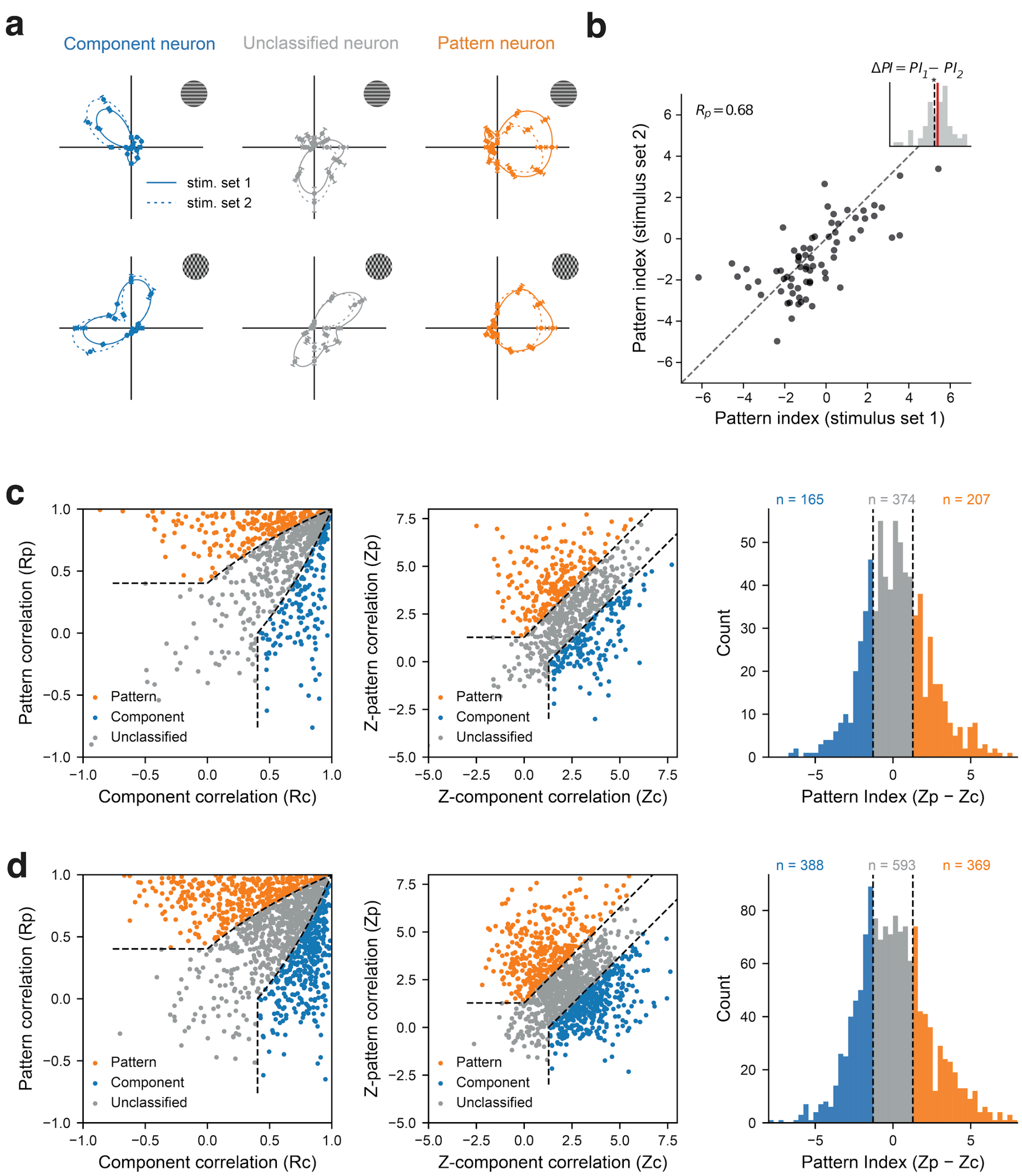

Supplementary Figure 7. Tuning to grating and plaid stimuli and classification of pattern and component neurons, split by stimulus set. (**a**) Tuning curves to gratings (top) and plaids (bottom) for three example neurons when presented with the two sets of component and pattern motion stimuli tested in this study in the same session (see **Fig. S6**). Solid lines denote stimulus set 1 and dashed lines denote stimulus set 2. Error bars denote SEM. (**b**) Correlation between the pattern index computed using stimulus set 1 versus stimulus set 2 in the same session. The Pearson correlation coefficient and identity line (dashed black) are denoted. Inset illustrates the pairwise difference in pattern index between the two stimulus sets, with mean difference denoted by the red line and 0 denoted by the dashed black line. Asterisk denotes significant difference from 0 (Wilcoxon signed-rank test, mean diff.=0.34, $p < 0.05$). **(c,d)** Classification of pattern and component neurons in sessions where stimulus set 1 (c) and stimulus set 2 (d) was presented. Conventions follow **Fig. 2c**.
